# In vitro efficacy of essential oils against *Sarcoptes scabiei*

**DOI:** 10.1038/s41598-022-11176-x

**Published:** 2022-05-03

**Authors:** Valérie Andriantsoanirina, Jacques Guillot, Michel Ratsimbason, Ghozlene Mekhloufi, Faliarivony Randriamialinoro, Lalasoanirina Ranarivelo, Frédéric Ariey, Rémy Durand

**Affiliations:** 1 Antiparasite Chemotherapy, BioCis, Université Paris Saclay, CNRS, 92290 Chatenay Malabry, France; 2 EA 7380 Dynamic, Ecole Nationale Vétérinaire d’Alfort, UPEC, USC ANSES, Maisons-Alfort, France; 3 Dermatology Parasitology Mycology Dept, ONIRIS, Ecole Nationale Vétérinaire, Agroalimentaire et de l’Alimentation, 44300 Nantes, France; 4 Centre National d’Application de Recherches Pharmaceutiques, Tananarive, Madagascar; 5 Institut Galien Paris-Saclay, CNRS, Université Paris-Saclay, 92290 Châtenay-Malabry, France; 6 INSERM U1016, Institut Cochin, Laboratoire de Parasitologie-Mycologie, Hôpital Cochin, AP-HP, Université de Paris, Paris, France; 7 Laboratoire de Parasitologie-Mycologie, Hôpital Henri Mondor, AP-HP, 1 rue Gustave Eiffel, 94000 Créteil, France

**Keywords:** Drug discovery, Diseases, Medical research

## Abstract

The mite *Sarcoptes scabiei* is responsible for scabies, a pruritic and contagious skin disease in humans. *S. scabiei* is also responsible for mange in a wide range of animal species. The treatment of *S. scabiei* infection is hampered by an under-effectiveness of the few available drugs. The objective of this work was to evaluate the in vitro acaricide activity of a large number of plant essential oils (EOs) against *S. scabiei*. EOs were selected mainly on the basis of traditional treatments for dermatological infections in Madagascar. The sarcoptes originating from a porcine animal model were tested at concentrations ranging from 10 to 0.1%. The viability of sarcoptes was assessed by stereomicroscopic observation at 5 min, 15 min, 30 min, 45 min and then every hour until 6 h after treatment. Estimates of lethal time and lethal concentration producing 50% mortality were generated using a probit analysis. The survival curves were estimated using the Kaplan Meier method. A total of 31 EOs from different plants were tested. *Cinnamomum zeylanicum* (cinnamom) and *Ocimum sanctum* (tulsi) oils were the most active for all concentrations tested. They may be included in in vivo studies, in order to further assess their potential interest as topical treatments.

## Introduction

*Sarcoptes scabiei* is an ectoparasite responsible for the emerging or re-emerging skin disease called scabies in humans and sarcoptic mange in animals. *S. scabiei* results in significant morbidity and mortality in wild and domestic mammals, with potential major economic loss. Scabies is a public health concern which affects more than 130 million people worldwide^1^. In humans, scabies is generally a nuisance due to its severe itching. Its main morbidity is related to secondary bacterial infections due to *Streptococcus pyogenes* and *Staphylococcus aureus*. The addition of scabies to the World Health Organization list of neglected tropical diseases in 2017 highlights the urgent need to develop better scabies control strategy^2^.

The treatment of scabies remains challenging, especially in tropical and subtropical areas^3^. In humans, current treatments of scabies rely on topical agents, permethrin 5% cream or benzyl benzoate 10–25%, or oral ivermectin^4^. However, cases of parasite resistance to some major scabicides such as pyrethroids and ivermectin are increasingly reported. It is therefore urgent to develop alternative approaches for the control of scabies^5–11^. In addition, mild to severe adverse effects of synthetic acaricides have been reported^7,12–14^.

As a consequence, alternative approaches to conventional acaricides are needed and plant essential oils (EOs) have been considered among other compounds. EOs are mixtures of volatile compounds which are usually extracted from aromatic plants through steam or hydro-distillation^15^. These compounds include two groups of distinct biosynthetical origin. The main group is composed of terpenes and terpenoids and the other of aromatic and aliphatic constituents, all characterized by low molecular weight. EOs can be obtained from different parts of plants, such as flowers, leaves, fruits, seeds, grasses and dried flower buds^16^. They are usually composed of 20–80 components, with the major molecules account for up to 85%, whereas other components are present in trace amounts. The effectiveness of EOs, mainly used topically for dermatological infections, against bacteria, fungi and parasites, has been demonstrated in several studies^17,18^. In a previous work, our team assessed the in vitro activity of ten EOs against *S. scabiei*. A limited number of other EOs were examined in other studies^19–21^. Among these EOs, a small number may have the potential to be included in acaricide formulations. This is already the case of tea tree oil which is included in a topical treatment combined with benzyl benzoate in a formulation available in Australia^19^. The aim of the present work was to study the in vitro acaricidal efficacy of a greater number of EOs. Most of these EOs were extracted from plants not previously considered for this purpose and originating from tropical countries, particularly from Madagascar.

## Materials and methods

### Essential oils

Essential oils of these plants were selected on the basis of historical reports on traditional treatments of dermatological infections in Indian Ocean area. Other EOs were chosen according to bibliographical data^22–26^. EOs were provided by the Centre National d’Application de Recherche Pharmaceutique, Tananarive, Madagascar, or were purchased for those commercially available (Table 1). EOs of two plants, *Psiadia altissima* and *Tetradenia nervosa*, extracted at CNARP Madagascar, are not wild or endemic plants and are not species at risk of extinction. The use of these plant species has complied with relevant institutional, national, and international guidelines and legislation.

**Table 1:** Plant species and essential oils tested.

Essential oil	Scientific name	Country	Extracted part
Lemon myrtle	*Backhousia citriodora*	Australia	Leafy branch
Rosalina	*Melaleuca ericifolia*	Australia	Leaf
Rosewood	*Aniba rosaeodora*	Brasil	Leaf
Coriander	*Coriandrum sativum*	Bulgaria	Seed
Camphorwood	*Cinnamomum camphora*	China	Leafy branch
Litsea	*Litsea citrata*	China	Fruits
Provence Cypress	*Cupressus sempervirens*	France	Leafy branch
Haitian vetiver	*Vetiveria zizanoides*	Haiti	Roots
Tulsi	*Ocimum sanctum*	India	Leaf
Patchouli	*Pogostermon cablin*	Indonesia	Leaf
Ahibero	*Cymbopogon giganteus*	Madagascar	Leaf
Bourbon Geranium	*Pelargonium asperum*	Madagascar	Leaf
Cinnamom	*Cinnamomum zeylanicum*	Madagascar	Leaf
Combawa	*Citrus hystrix*	Madagascar	Leaf
Eucalyptus citriodora	*Eucalyptus citriodora*	Madagascar	Leaf
Iary	*Psidia altissima*	Madagascar	Flowering tops
Katrafay	*Cedrelopsis grevei*	Madagascar	Bark
Lantana	*Lantana camara*	Madagascar	Flowering tops
Niaouli	*Melaleuca quinquenervia*	Madagascar	Leaf
Ravintsara	*Cinnamomum camphora*	Madagascar	Leaf
Saro	*Cinnamosma fragrans*	Madagascar	Leaf
Tagetes	*Tagetes minuta*	Madagascar	Flowering tops
Turmeric	*Curcuma longa*	Madagascar	Roots
Turpentine	*Pinus pinaster*	Madagascar	Resins
Rambiazina	*Helichrysum gymnocephalum*	Madagascar	Flowering tops
Tetradenia	*Tetradenia nervosa*	Madagascar	Leaf
Ylang ylang	*Cananga odorata*	Portugal	Flowers
Cryptomeria	*Cryptomeria japonica*	Reunion Island	Leafy branch
Lime	*Citrus limonum*	Sicily	Zest
Eucalyptus globulus	*Eucalyptus globulus*	Spain	Leaf
Myrtle	*Myrtus communis*	Tunisia	Leafy branch

EOs were prepared for assays by diluting them in paraffin oil which was also used as control. The dilutions were performed right before setting for contact or fumigation bioassays.

### Collection of mites

*S. scabiei* mites were collected from infected pigs maintained at the Centre de Recherche Bio-Médicale, Maisons-Alfort, France. Pigs were experimentally infested as previously described^27^. All animals were maintained in strict accordance with good animal practices as defined by the French and European code of practice for the care and use of animals for scientific purposes (approval No. 02515.01). The study protocol was approved by the « Comité d’éthique en expérimentation animale Anses/EnvA/UPEC » (the local ethics committee for animal experiments). The study was carried out in compliance with the ARRIVE guidelines. Mites collected from the ears and back of pigs were placed in Petri dishes. All Petri dishes were incubated at 35 °C at least 30 min before experiments. Mites were observed under a stereomicroscope (Nikon©, SMZ645, Lisses, France) and motile stages (adults and larvae) were included in the assays.

### Contact bioassays

The acaricide activity of the EOs was evaluated by bioassays as described by Fang et al. with slight modifications^28^. In a first screening, all EOs were diluted with paraffin oil at 10% and 5%. The evaluation was conducted further for EOs showing acaricidal activity in contact bioassays in less than 3 h at the 5% concentration. These EOs were then diluted with paraffin oil at 1%, 0.5% and 0.25% concentrations.

For each experiment, adult and larvae stages (n = 10) were collected with a needle and placed directly in plastic Petri dish (5 cm in diameter) in direct contact with 50 µl of each diluted solution. All experiments were done in triplicate. Negative control mites were treated with paraffin oil and clove essential oil was used as positive control as previously described^28^. All dishes were incubated at room temperature and observed under a stereomicroscope at 5 min, 15 min, 30 min, 45 min then every hour to 6 h post-treatment. The mites were regularly stimulated with a needle to determine their viability. When they did not respond to stimulation, they were recorded as dead.

### Fumigation bioassays

The vapor phase toxicity of all EOs was investigated. For each fumigation bioassay, 10 mites of motile mixed stages were placed at the bottom of a plastic Petri dish (5 cm in diameter). A covering filter paper (Whatman, No. 2,5 cm in diameter) was put on the lid of the Petri dish and treated with 200 µl of EOs diluted in paraffine oil at concentration of 5% and 1%. Negative control received paraffin oil alone and tea tree oil was used as a positive control as described by Fang et al*.*^28^. Three replicates were performed for each concentration. Mortality was recorded after 3 h. The mites were stimulated with a needle to determine their viability. When they did not respond to stimulation, they were recorded as dead.

### Ovicidal activity

*Cinnamomum zeylanicum* and *Ocimum sanctum* 5% oils were selected for evaluating their ovicidal activity. Scabies eggs were counted into groups of 10 and were placed directly in the bottom of Petri dish (5 cm in diameter) with 50 µl of each solution or paraffin oil for control. The dishes were incubated at 30 °C under 75% relative humidity for 5 days. The number of hatched and unhatched eggs were counted under microscope at the end of the 5-d period.

### Gas chromatography/Mass spectrometry analysis

The main components of the EOs were analyzed by gas chromatography/mass spectrometry with an Agilent 5977A Series GC/MSD System as previously described^28^.

### Statistical analyses

Probit regression estimates and lethal time and concentration producing 50% mortality in all stages were generated using probit analysis^29^. Survival curves were estimated using the Kaplan Meier method. The 95% CI were calculated using a normal distribution. Difference in the survival curves were assessed using the Log-rank test adjusted with Tukey’s method. The median lethal time (LT_50_ values) of EOs was calculated using a generalized linear model with binomially distributed error term. R v 3.5.1 and SAS v9.4 were used for all analyses. The significant level was set at *p* < 0.05.

### Ethical approval

Mites were collected from experimentally infected pigs which were maintained in strict respect to the guidelines as defined by the French and European code of good practice for the care and use of animals for scientific purposes (French Ministry of Research approval no: 0251503).

## Results

**Figure 1:**
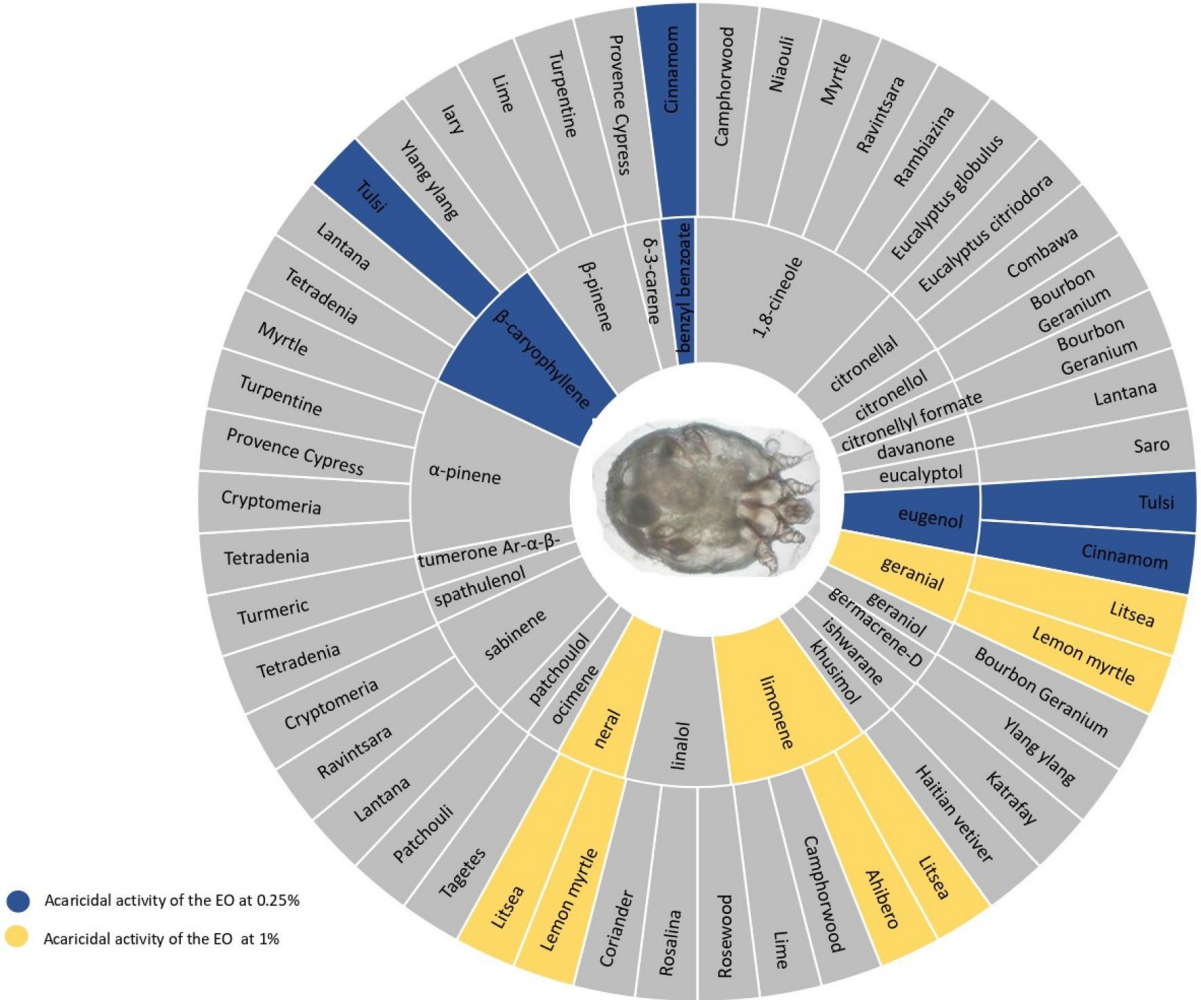
Sunburst display of the main components of the 31 essential oils.

The principal components of EOs used in this study are shown in Fig. 1.

**Figure 2:**
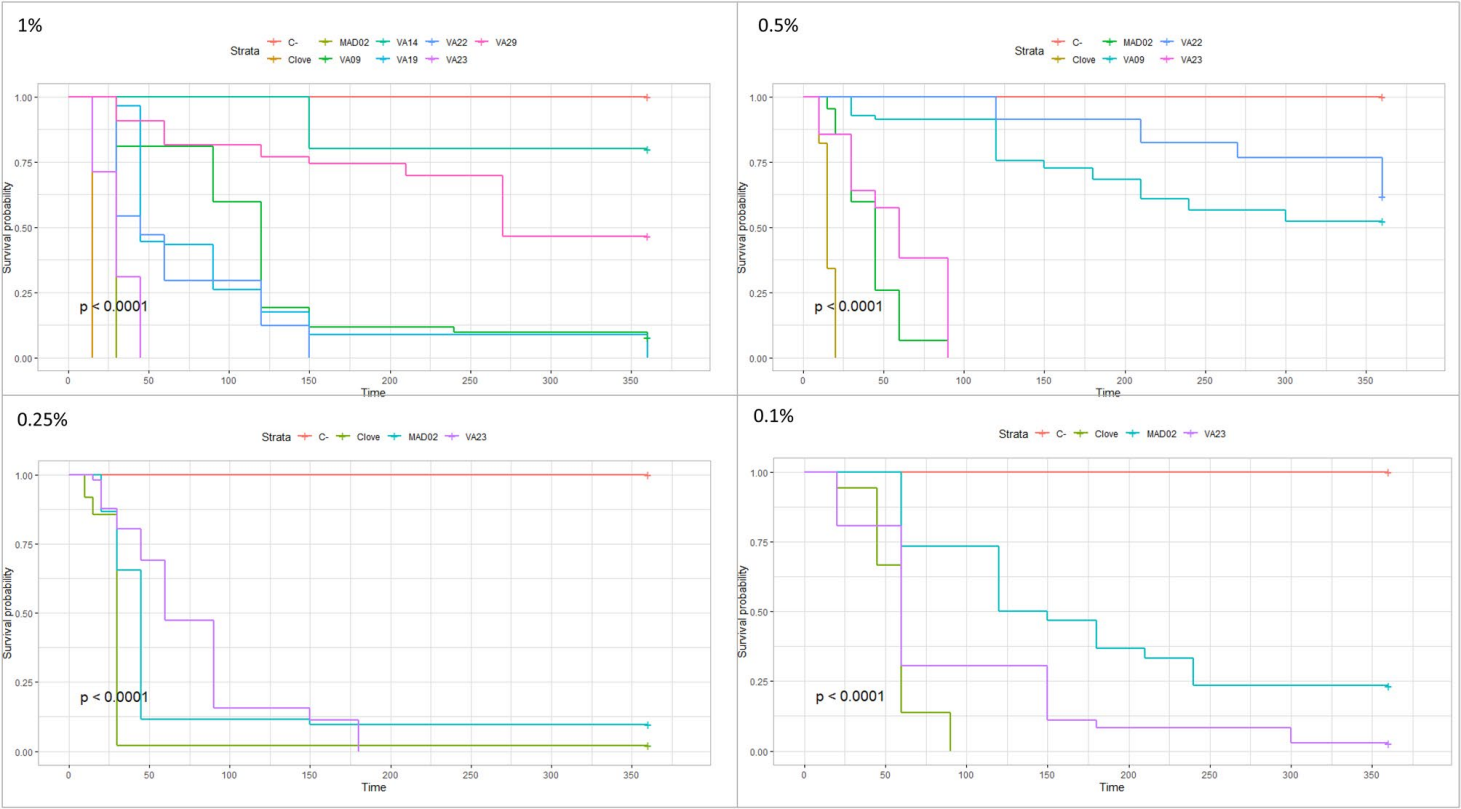
Survival curves of sarcoptes exposed to essential oils at 0.1%, 0.25%, 0.5%, and 1% concentrations in contact assay (C : paraffin oil).

There was no mortality of *S. scabiei* in paraffin oil. The survival curves for the different concentrations are shown in Fig. 2.

 In pre-screening of all EOs, in contact bioassays, only four EOs (katrafay, turmeric, turpentine and lantana) showed no acaricidal property at 10% concentration (Table 2).

 At 5% concentration, 14 out 31 EOs showed acaricidal property. Five EOs were still effective at 1% concentration. Two Eos (tulsi and cinnamom) remained acaricidal at 0.5% concentration (and even at 0.25% concentration for tulsi oil). The LT_50_ values of EOs tested at 1%, 0.5% and 0.25% concentrations are shown in Table 3.

**Table 2:** Acaricidal activity determined by contact and fumigation bioassays for 31 essential oils at different concentrations (10–0.25%).

	Essential oils		Contact activity (%)					Fumigant activity (%)	
			10	5	1	0.5	0.25	5	1
*Ocimum sanctum*	Tulsi	VA23	+	+	+	+	+	+	+
*Cinnamomum zeylanicum*	Cinnamom	MAD 02	+	+	+	+	±	+	+
*Pelargonium asperum*	Bourbon geranium	VA14	+	+	+	−	−	+	±
*Litsea citrata*	Litsea	VA19	+	+	+	−	−	+	±
*Backhousia citriodora*	Lemon myrtle	VA22	+	+	+	-	−	+	±
*Cymbopogon giganteus*	Ahibero	VA09	+	+	±	−	−	+	+
*Aniba rosaeodora*	Rosewood	VA29	+	+	±	−	−	+	±
*Cinnamosma fragrans*	Saro	VA02	+	+	−	−	−	−	−
*Cinnamomum camphora*	Ravintsara	VA03	+	+	−	−	−	−	−
*Eucalyptus citriodora*	Eucalyptus citriodora	VA06	+	+	−	−	−	+	−
*Cananga odorata*	Ylang ylang	VA08	+	+	−	−	−	−	−
*Cinnamomum camphora*	Camphorwood	VA26	+	+	−	−	−	+	±
*Melaleuca ericifolia*	Rosalina	VA27	+	+	−	−	−	+	±
*Coriandrum sativum*	Coriander	VA28	+	+	−	−	−	+	±
*Tetradenia nervosa*	Tetradenia	T NERV	+	−	−	−	−	−	−
*Psiadia altissima*	Iary	P ALT	+	−	−	−	−	−	−
*Tagetes minuta*	Tagete	VA11	+	−	−	−	−	−	−
*Melaleuca quinquenervia*	Niaouli	VA12	+	−	−	−	−	±	−
*Citrus hystrix*	Combawa	VA13	+	−	−	−	−	+	−
*Cupressus sempervirens*	Provence cypress	VA15	+	−	−	−	−	−	−
*Vetiveria zizanoides*	Haitian vetiver	VA16	+	−	−	−	−	−	−
*Cryptomeria japonica*	Cryptomeria	VA17	+	−	−	−	−	−	−
*Eucalyptus globulus*	Eucalyptus globulus	VA18	+	−	−	−	−	−	−
*Helichrysum gymnocephalum*	Rambiazina	VA20	+	−	−	−	−	±	−
*Myrtus communis*	Myrtle	VA21	+	−	−	−	−	−	−
*Pogostermon cablin*	Patchouli	VA24	+	−	−	−	−	−	−
*Citrus limonum*	Lime	VA25	+	−	−	−	−	−	−
*Cedrelopsis grevei*	Katrafay	VA04	−	−	−	−	−	−	−
*Curcuma longa*	Turmeric	VA05	−	−	−	−	−	−	−
*Pinus pinaster*	Turpentine	VA07	−	−	−	−	−	−	−
*Lantana camara*	Lantana	VA10	−	−	−	−	−	−	−

( +) : all sarcoptes died ; (−) : all sarcoptes alive ; ( ±) : sarcoptes alive but motionless.

**Table 3:** LT_50_ values measured by minute for all stages of sarcoptes (*most of sarcoptes died before 30 min).

LT_50_ (min)								
Essential oils	1%	95% CI	0.5%	95% CI	0.25%	95% CI	0.1%	95% CI
Clove	17.50*	–	18.02	[16.79–19.15]	30.82	[27.00– 33.96]	63.06	[55.80–69.17]
Cinnamom	37.53*	–	40.15	[35.12–44.32]	42.19	[38.63–45.09]	88.35	[39.85–115.19]
Tulsi	23.54	[8.15–35.11]	28.15	[21.83–32.83]	42.64	[26.64–54.92]	92.97	[66.36–110.42]
Ahibero	62.53	[33.39–82.21]	–	–	–	–	–	–
Lemon myrtle	35.15	[21.00–45.97]	–	–	–	–	–	–
Litsea	32.60	[6.34–50.85]	–	–	–	–	–	–

All mites were killed within one hour with tulsi oil and cinnamom oil diluted at 1%. The cinnamom and tulsi oils exhibited also the highest percentage of mortality at 0.25% concentration over an exposure period of 6 h. Tulsi oil and cinnamom oil were the most active oils for all concentrations tested, followed by lemon myrtle, litsea, bourbon geranium, rosewood and ahibero.

The results of the fumigation assay were not completely in accordance with those of the fumigation assay, though most effective EOs in contact assays were also the most effective in fumigation assays (Table 2). Among EOs which were not acaricide at 5% concentration in contact assays, only combawa showed efficacy by fumigation at 5% concentration. Interestingly, tulsi and cinnamom, showed equal efficacy by contact and fumigation. Nevertheless, these two EOs demonstrated no ovicidal activity in comparison with the negative control group (Data not shown).

## Discussion

We studied EOs originating from various plants selected on the basis of their current or past use in traditional medicine. Local and traditional knowledge has been the starting point for many successful drug development projects in the past. Such approach is generally based on a detailed observation of how local populations use plants. Quinine and artemisinine derivatives, used as antimalarial treatments, are famous examples of this successful approach in tropical medicine. Madagascar and more largely Indian Ocean islands are renowned for their large biodiversity, either animal or vegetal, with the presence of many endemic species. Traditional medicine is also well developed in these areas. Scabies can manifest with a wide spectrum of clinical signs and variable severities, making clinical diagnosis difficult even for a medical staff. The condition is not fully recognized or characterized among indigenous populations. Thus, in our search for potentially interesting plants in the literature, we selected a large acceptation of terms such as “pruritus” or “dermatological affection”.

Results obtained in vitro with tested EOs are not necessarily reproducible from one study to another. EOs may vary in the nature and the concentration of their compounds within a certain range. These variations can be attributed to factors that influence the plant’s environment, such as climate, geographical location, soil type and fertilizer used^30,31^ and also to the chosen method of extraction. Thus, it is important to analyze the composition of the EOs tested in order to characterize the efficacy of most active EOs. This approach permits to identify the major components which are probably responsible for most of the effects of the EOs. Only two EOs of our series have been previously tested: one obtained from *Pelargonium asperum*^28^ and another one from *Citrus limonum*^32^. While the EO from *Pelargonium asperum* showed one of the best efficacies of our series, that from *Citrus limonum* was one of the less effective. These results were consistent with those of the previous studies^28,32^. Not surprisingly, citronellol was the major component of the EO from *Pelargonium asperum* found in both our study and that of Fang et al. Similarly, limonene was the main component found in the EO from *Citrus limonum* of our series and of that of other studies.

The biological properties of essential oils are usually determined by their major components^15,28,32^. Various major components were found in the present study according to the EOs tested. In particular, geranial and neral were the main components of lemon myrtle and litsea which were among the most effective EOs tested. Geranial and neral were also the main components of the lemongrass (*Cymbopogon citratus*) oil which has been reported as a promising agent for scabies control^21^. Likewise, eugenol was found as the main component of tulsi and cinnamom EOs, the most effective EOs of the present series. Eugenol was also the main component of clove oil which was found as the most scabicide agent in a previous, though more limited, series^28^. The present results confirmed the potential interest of geranial, neral and eugenol as scabicide agents. Actually, a drug containing eugenol (at a 2.5% concentration) is already on the Chinese market for scabies treatment since 1990s^33^.

Cinnamom oil has never been tested on *S. scabiei*. Its efficacy against *Psoroptes* mites, major parasites of ruminants or rabbits, has been demonstrated in vitro and in vivo studies^34^. The sample of Cinnamom oil used in the present study contained, besides eugenol, its majoritory compound, a high concentration of benzyl benzoate. Thus, it is not surprising that it appeared among the most effective EOs tested.

As previously reported^28^ the fumigant effect of EOs did not correspond exactly to their efficacy by direct contact. In the present study, 1,8-cineole, contained in several EOs, appeared more active by fumigation than by contact. This has been already shown as 1,8-cineole was the main component of eucalyptus oil^28^. Linalol, a major component of rosalina, coriander and camphorwood, and citronellal, a major component of combawa and eucalyptus citriodora, appeared also more active by fumigation than by contact in the present study. To our knowledge, this was not previously described.

We chose to study the ovicidal efficacy only for the most active EOs of our series on the basis of contact and fumigation assays because a compound having only ovicidal efficacy would probably be of limited therapeutic potential. Unfortunately, tulsi and cinnamom EOs showed no significant ovicidal efficacy. It may be underlined that current first line scabicides have no ovicidal activity either. Accordingly, potential formulations including these EOs with a therapeutic purpose would require two applications separated by a 10–14-day interval as it is the case for most current treatments.

We chose to use paraffin oil (mineral oil) to perform the dilutions of EOs and as treatment for negative control groups. Paraffin oil was the solvent the most frequently used as diluent for evaluating the effects of EOs against *Sarcoptes* mites in previous studies^20,21,28,35,36^. It has been demonstrated that paraffin oil has no toxicity against mites.

EOs are generally well tolerated as demonstrated by their millenary use in aromatherapy^37^. They are reported as slightly toxic’ or ‘non-toxic’, with an oral and dermal LD_50_ (Median lethal dose) value of 1–5 g/kg or more than 5 g/kg to mammals^38^. Understandably, the toxicity depends on the nature of the components and on their concentrations^38^. The EOs showing best scabicide efficacy by contact and fumigation assays, tulsi and cinnamom, would probably be well tolerated at concentrations ≤ 1% for topical use, though an allergic contact dermatitis may occur independently of the concentration.

Scabies affects often young children, particularly children aged 1–24 months^39^. However, first line treatments, permethrin and ivermectin, are not recommended for use in young children^40^. Some EOs within a certain range of dilution rates could be used in young children. Some dilution rates of EOs have been proposed according to age groups : 0.10% to 0.20% in full-term infant, aged 0–3 months ; 0.25–0.50% for 3–24 month-old children ; and 1–2% for 2–6 year-old children^38^. Tulsi and cinnamom EOs appeared effective in vitro within these ranges of dilution.

Owing to their multiple active components, EOs are less likely to lead to resistance comparing to conventional drugs^19^. In addition to their scabicide property, EOs may have various biological properties including antibacterial, anti-inflammatory, and antipruritic effects^16^. All these adjuvant properties are particularly interesting for the treatment of scabies.

In conclusion, there has been a growing body of evidence indicating the potential value of EOs as control agents against a range of ectoparasites, including *S. scabiei*. The present study demonstrated the contact and fumigant effects of new EOs which may be useful to incorporate in novel topical formulations for scabies control. Our results confirmed the scabicide efficacy by contact of some components as eugenol, geranial and neral. Other components as linalol and 1,8-cineole appeared more effective by fumigation. Two EOs, tulsi and cinnamom, proved to be more acaricide than others, though they appeared to be devoid of ovicidal activity. These two EOs may be tested in vivo, possibly using a pig or rabbit model to further evaluate their potential interest as topical treatments.

## Supplementary Information

Supplementary Information 1.

Supplementary Information 2.

Supplementary Information 3.

## Data Availability

The datasets supporting the conclusions of this article are included within the article. Raw data are available from the corresponding author on reasonable request.

## Abbreviations

EO: Essential oil
95% CI: Confidence interval 95%
LT_50_: Median lethal time
LD_50_: Median lethal dose
