# Supplementary material for: In vitro efficacy of essential oils against Sarcoptes scabiei
Source: Sci Rep. 2022 May 3;12:7176. doi: 10.1038/s41598-022-11176-x (PMC9065015; doi:10.1038/s41598-022-11176-x)
Supplement: Supplementary file 3 — Supplementary Information 3. [file 41598_2022_11176_MOESM3_ESM.docx]

Figure title: Composition of the most effective essential oils determined by gas chromatography/ mass spectrometry

Legend: VA23, VA19, VA22, VA29, VA09 and MAD02 are the name of the most effective essential oils samples used in the contact and fumigation assays. Main compounds (in %) are highlighted in yellow.

**VA23:** *Ocimum sanctum*

| Retention times (min) | Qualitative composition | Relative compositions (%) |
| --- | --- | --- |
| 49.9 | Geraniol | 3.3 |
| 65.8 | unknown | 0.4 |
| 67.6 | unknown | 0.6 |
| 73.5 | Elemol | Not detected |
| 76.3 | Eugenol | 54.5 |
| 77.5 | Beta-caryophyllene | 35.6 |
| 79.5 | Alpha-humulene | 5.0 |
| 89.3 | unknown | 0.6 |

*Chromatogram resulting from analysis of sample VA23 by GC-MS*

**
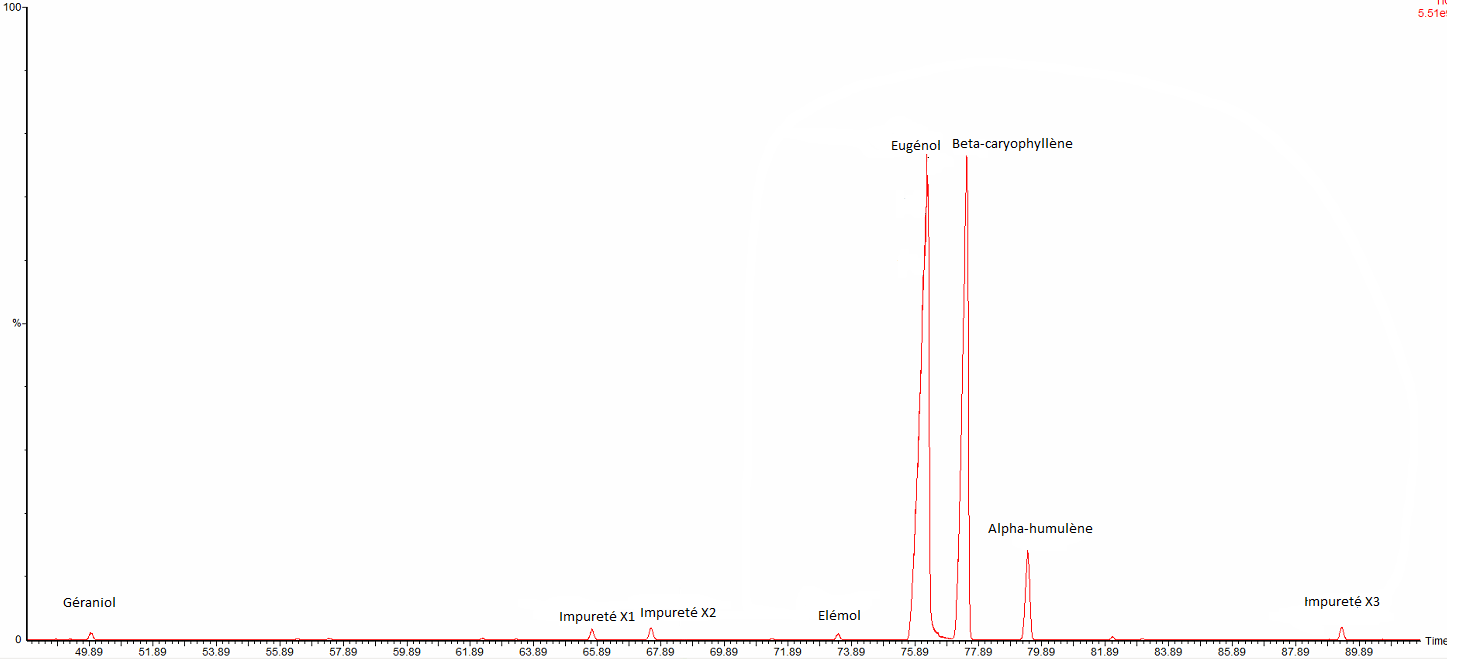
**

*Chromatogram from the analysis of sample VA23*

**VA19:** *Litsea citrata*

| Retention times (min) | Qualitative composition | Relative compositions (%) |
| --- | --- | --- |
| 40.82 | unknown | 1.21 |
| 42.54 | unknown | 0.21 |
| 44.94 | unknown | 0.75 |
| 47.99 | unknown | 0.87 |
| 48.89 | Limonene | 13.38 |
| 49.84 | unknown | 0.82 |
| 56.22 | unknown | 1.34 |
| 60.19 | unknown | 1.55 |
| 60.86 | unknown | 0.75 |
| 62.08 | unknown | 1.36 |
| 63.79 | unknown | 0.34 |
| 66.05 | unknown | 0.99 |
| 68.21 | Neral | 32.26 |
| 70.20 | Geranial | 43.20 |
| 77.15 | unknown | 0.96 |


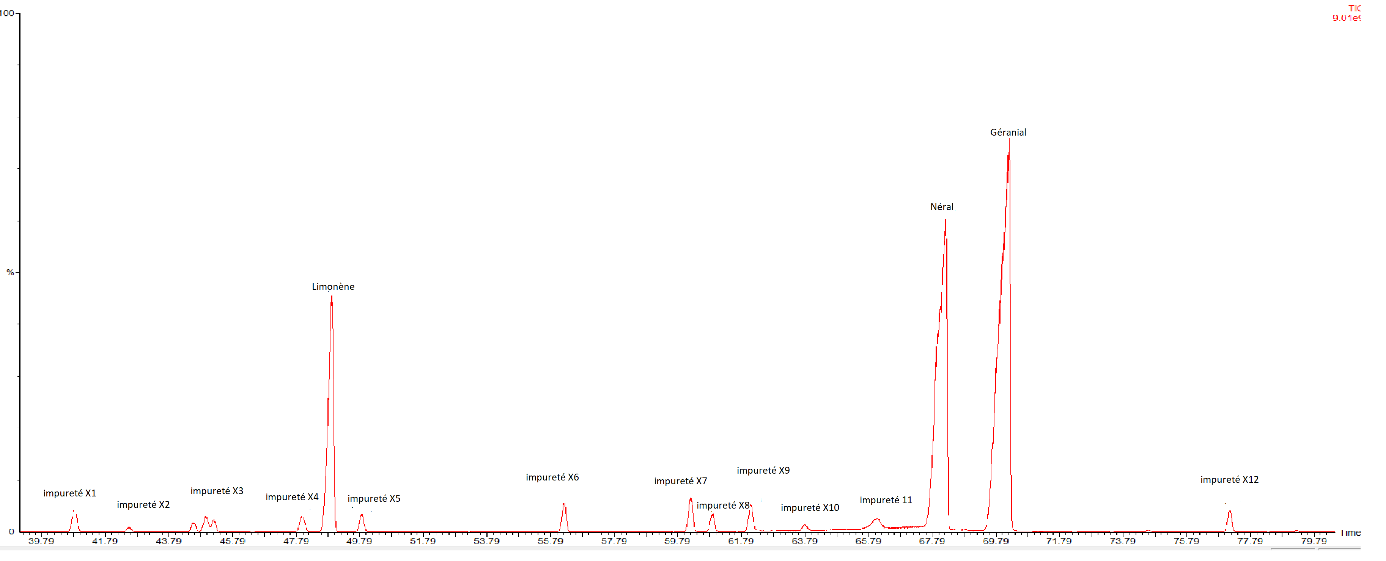


*Chromatogram from the analysis of sample VA19*

**VA22 :** *Backhousia citriodora*

| Retention times (min) | Qualitative composition | Relative compositions (%) |
| --- | --- | --- |
| 45.11 | unknown | 0.06 |
| 48.11 | unknown | 0.82 |
| 48.89 | unknown | 0.74 |
| 56.35 | Linalol | 0.60 |
| 60.35 | unknown | 0.56 |
| 61.05 | unknown | 0.31 |
| 62.22 | unknown | 0.58 |
| 68.41 | Neral | 36.97 |
| 70.53 | Geranial | 58.04 |
| 77.28 | unknown | 1.32 |


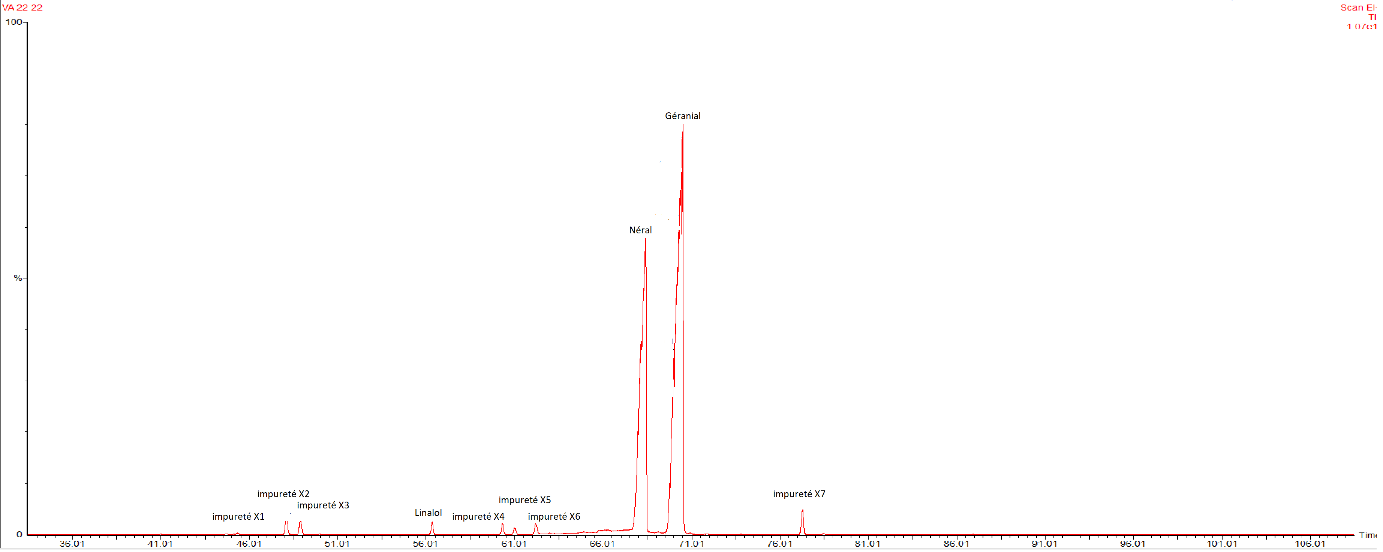


*Chromatogram from the analysis of sample VA22*

**VA29 :** *Aniba rosaeodora*

| Retention times (min) | Qualitative composition | Relative compositions (%) |
| --- | --- | --- |
| 48.89 | unknown | 0.09 |
| 50.00 | Geraniol | 0.10 |
| 54.50 | Cis-oxide linalol | 0.74 |
| 55.60 | Trans-oxide linalol | 0.61 |
| 56.75 | Linalol | 97.17 |
| 63.90 | Alpha-terpineol | 0.28 |
| 73.47 | unknown | 0.29 |
| 81.21 | unknown | 0.73 |


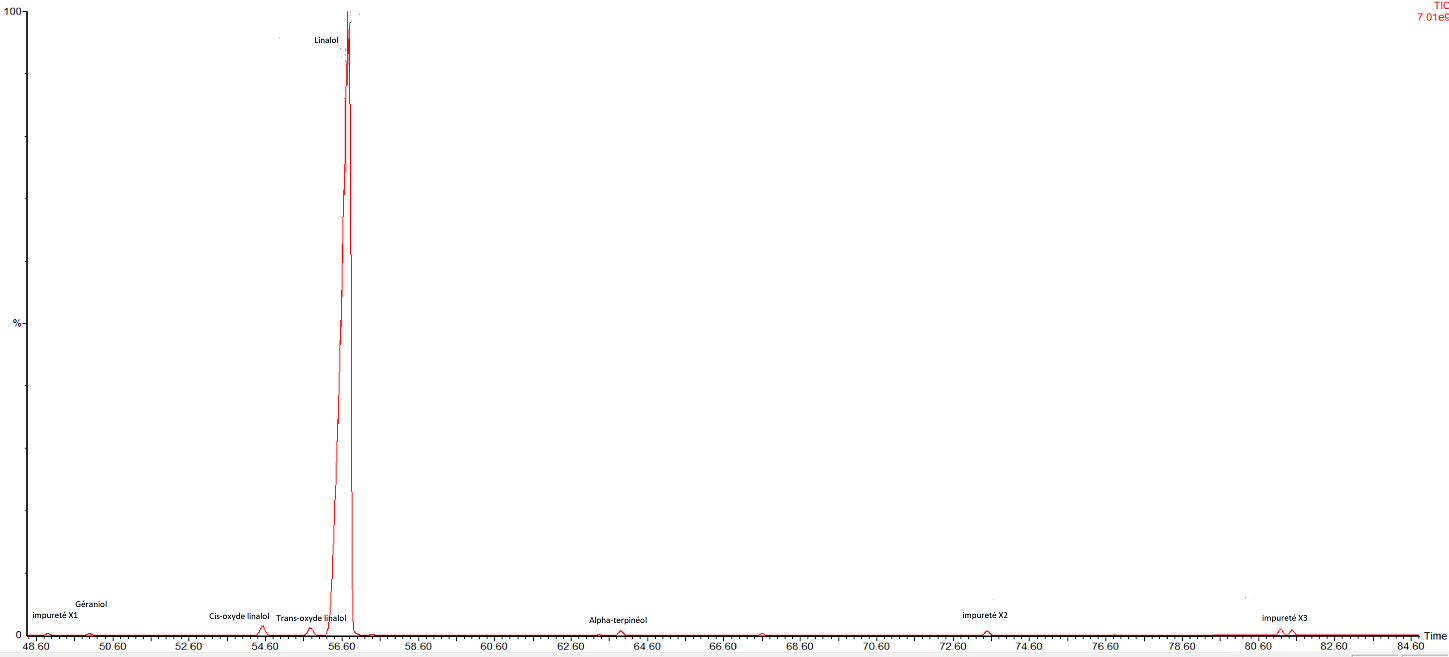


*Chromatogram from the analysis of sample VA29*

**VA09 :** *Cymbopogon giganteus*

| Retention times (min) | Qualitative composition | Relative compositions (%) |
| --- | --- | --- |
| 48.98 | Limonene | 30.17 |
| 59.09 | Trans-p-Mentha-1.8-dien-2-ol | 21.95 |
| 60.24 | Cis-p-Mentha-2.8-dien-1-ol | 7.84 |
| 62.91 | unknown | 1.16 |
| 64.20 | Trans-p-Mentha-2.8-1-ol | 16.25 |
| 64.74 | unknown | 1.47 |
| 66.1 | unknown | 1.39 |
| 66.26 | unknown | 1.37 |
| 67.37 | Cis-p-Mentha-1.8-2-ol | 15.89 |
| 68.42 | unknown | 2.51 |


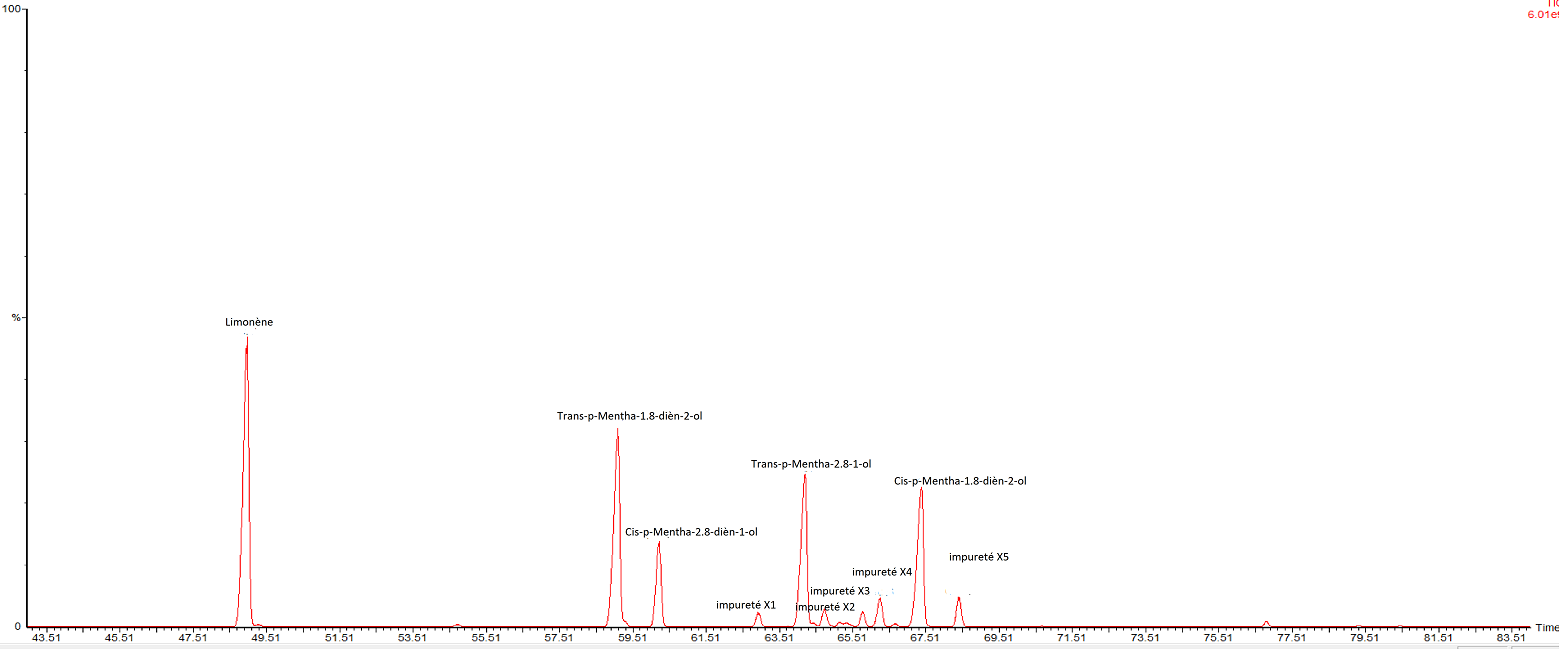


*Chromatogram from the analysis of sample VA09*

**MAD02** : *Cinnamomum zeylanicum*

| Retention times (min) | Qualitative composition | Relative composition % |
| --- | --- | --- |
| 49.27 | unknown | 0.88 |
| 56.19 | Linalol | 0.90 |
| 71.84 | unknown | 3.26 |
| 76.5 | Eugenol | 53,36 |
| 77.29 | Beta-caryophyllene | 3.58 |
| 80.25 | Alpha-humulene | 2.65 |
| 85.60 | unknown | 5.32 |
| 99.12 | Benzyl benzoate | 30.04 |


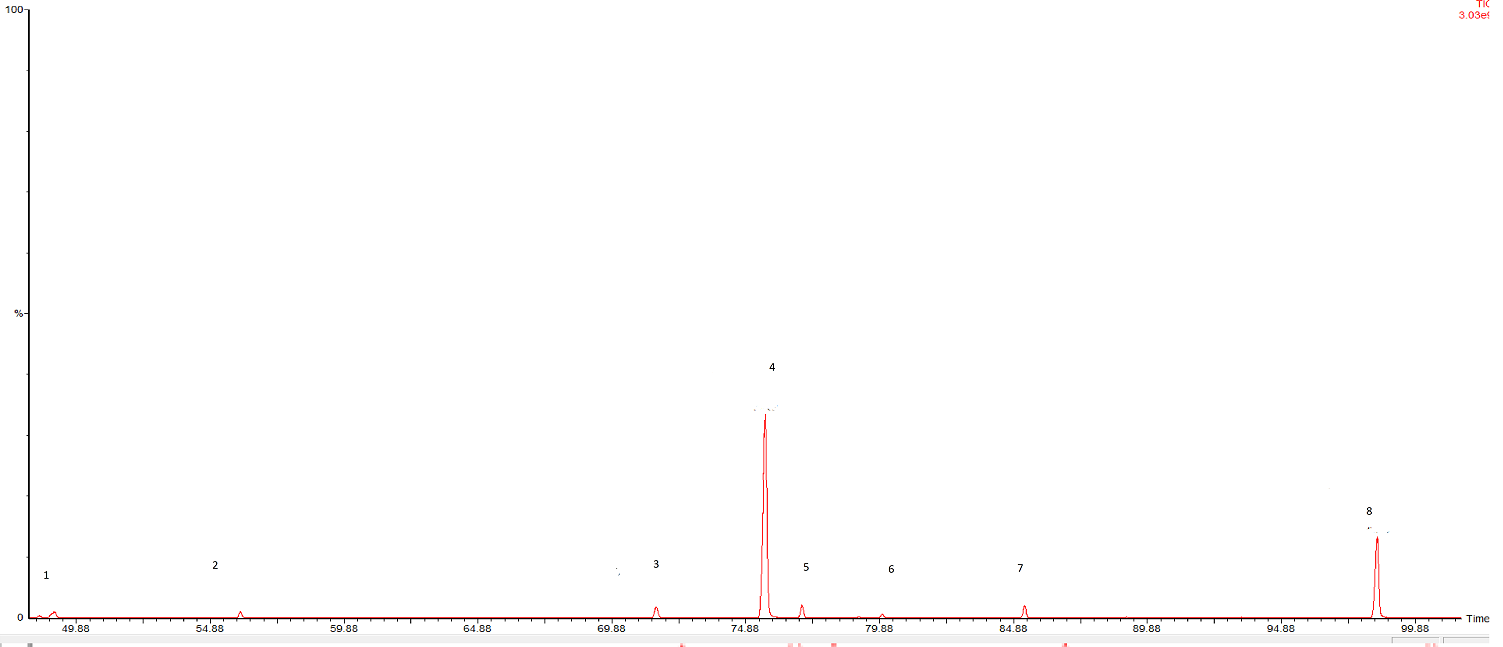


*Chromatogram from the analysis of sample MAD02*
